# A Manual of the Practice of Medicine

**Published:** 1899-04

**Authors:** 


A MANUAL OF THE PRACTICE OF MEDICINE. By FREDERICK TAYLOR, M. D., F. R. C. P., Physician to, and Lecturer on, Medicine at Guy's Hospital, etc. Fifth edition. Pp. 1,002. London: J. & H. Churchill, 7 Great Marlborough Street. Philadelphia: P. Blakiston's Son & Co. 1898.

The fifth edition of this standard text-book finds it carefully revised, some sections largely rewritten and notices of many diseases recently recognised or omitted from former editions added. The introduction to the Diseases on the nervous system has been revised and partially rewritten and chapters added, especially on aphasia, erythromelalgia, angeioneurotic edema, Diver's palsy, and yet these chapters could very well be left to the special works on nervous diseases, where they would receive the space and attention which they deserve.

A separate section has been given to diseases of the mediastinum, and filarial disease and hemoglobinuria have been transferred to diseases of the lymphatic system and the blood respectively. The style of the printing of the work has also been changed, with the larger page and clearer type; the value of the book to students and practitioners is greatly enhanced. W. C. K.

